# Effects of continuous positive airway pressure and mandibular advancement appliance therapy on sleep bruxism in adults with obstructive sleep apnea: a pilot study

**DOI:** 10.1007/s11325-023-02799-z

**Published:** 2023-03-03

**Authors:** Deshui Li, Frank Lobbezoo, Boyuan Kuang, Antonius A. J. Hilgevoord, Nico de Vries, Ghizlane Aarab

**Affiliations:** 1 Department of Orofacial Pain and Dysfunction, Academic Centre for Dentistry Amsterdam (ACTA), University of Amsterdam and Vrije Universiteit Amsterdam, Amsterdam, The Netherlands; 2 Department of Clinical Neurophysiology, OLVG, Amsterdam, The Netherlands; 3 Department of Orthodontics, School and Hospital of Stomatology, Shandong University, Jinan, China; 4 Taikang Bybo Dental, Shanghai, China; 5 Department of Otorhinolaryngology, OLVG, Amsterdam, The Netherlands; 6 Faculty of Medicine and Health Sciences, Department of Otorhinolaryngology, Head and Neck Surgery, Antwerp University Hospital (UZA), Edegem, Belgium

**Keywords:** Obstructive sleep apnea, Sleep bruxism, Polysomnography, Continuous positive airway pressure, Mandibular advancement appliance

## Abstract

**Study objectives:**

This study aimed to investigate the effects of continuous positive airway pressure (CPAP) and mandibular advancement appliance (MAA) therapy on rhythmic masticatory muscle activity (RMMA), a biomarker of sleep bruxism (SB), and to compare the effects of CPAP with MAA in adults with obstructive sleep apnea (OSA).

**Methods:**

This cohort study included individuals with OSA who received treatment with CPAP or MAA. Polysomnographic recordings with and without therapy were performed in each individual. Statistical analyses were performed with repeated measures ANOVA.

**Results:**

A total of 38 individuals with OSA were enrolled, 13 on CPAP and 25 with MAA, mean age 52.6 ± 10.6 years, 32 men, mean baseline apnea-hypopnea index (AHI) 26.5 ± 15.2 events/hour, mean RMMA index 3.5 ±events/hour. In the total group, the RMMA index decreased significantly with CPAP and MAA therapies (*P* < 0.05). The changes in the RMMA index with therapy did not differ significantly between CPAP and MAA (*P* > 0.05). The RMMA index decreased in 60% of the individuals with OSA, and the changes ranged widely, with a median of 52% and an interquartile range of 107%.

**Conclusions:**

Both CPAP and MAA therapies significantly reduce SB in individuals with OSA. However, the interindividual differences in the effects of these therapies on SB are large.

**Clinical Trial Registration:** https://trialsearch.who.int (NL8516); April 08, 2020

## Introduction

Obstructive sleep apnea (OSA) is a sleep-related breathing disorder characterized by apneic (absent airflow) or hypopneic (reduced airflow) events that result from repetitive narrowing and/or collapsing of the upper airway and that commonly result in oxygen desaturations and arousals from sleep [1]. Individuals with OSA commonly have symptoms of morning headache, daytime sleepiness, and loud snoring [1]. The prevalence of OSA ranges from 2 to 14% in community-screened populations to a higher prevalence in certain subgroups (*e.g.,* in men and in obese individuals) [2, 3]. OSA has been reported to be associated with many other health conditions such as stroke, hypertension, depression, diabetes, coronary artery disease, and sleep-related movement disorders, such as periodic leg movement during sleep and sleep bruxism (SB) [4–6].

SB is a masticatory muscle activity during sleep that is characterized by rhythmic masticatory muscle activity (RMMA) and may induce severe tooth wear, orofacial pain, and temporomandibular disorder [7]. Previous study has reported that OSA could be an independent risk factor for SB [8]. Furthermore, some studies reported that around half of individuals with OSA also have SB, suggesting that SB is a common comorbidity of OSA and that there is a close association between the two conditions [9, 10].

Although the underlying mechanism of the association between OSA and SB is still unclear, previous studies suggested that the occurrence of RMMA might be related to recurrent respiratory events and sleep arousals in OSA. Based on this, we hypothesized that effective OSA therapies will decrease the frequency of RMMA in individuals with OSA. This has been reported in two cases with concomitant OSA and SB [11, 12]. RMMA episodes in these two cases disappeared during continuous positive airway pressure (CPAP) therapy, while RMMA recurred when CPAP was removed [11, 12]. Similarly, other studies have demonstrated that mandibular advancement appliances (MAA) significantly reduced RMMA episodes in individuals with SB [13, 14]. However, few studies have been performed to investigate the effects of OSA therapies on SB in adults with OSA. Therefore, the first aim of this pilot study was to determine the effects of OSA therapies (CPAP and MAA) on SB in adults with OSA and in a subgroup of those with comorbid SB. We hypothesized that both CPAP and MAA therapies will significantly reduce RMMA in adults with OSA as well as in those with comorbid SB. In addition, since a growing number of studies indicated that the occurrence of RMMA was related to arousals rather than to respiratory events [15, 16], we hypothesized that both OSA therapies would significantly reduce RMMA related to respiratory arousals (RMMA-RAr), while having no effect on RMMA related to non-respiratory arousals (RMMA-nRAr). Therefore, the second aim of this pilot study was to investigate the effect of CPAP and MAA on the RMMA-RAr and RMMA-nRAr in individuals with OSA and in those with comorbid SB.

## Methods

This cohort study is part of a large-scale prospective polysomnographic (PSG) study on the associations between SB and other sleep-related disorders. The protocol was approved by the institutional Medical Ethics Committee of the OLVG West, Amsterdam (WO 16–577). This study has also been registered on trialsearch.who.int (Trial NL8516).

### Participants

The sample was collected on the basis of the medical history of patients who were referred to the Department of Clinical Neurophysiology, OLVG West, Amsterdam, the Netherlands, between April 2017 to July 2018. Individuals who met the following criteria were included in this study: 1) age ≥ 18 years; 2) diagnosed with OSA (baseline apnea–hypopnea index (AHI) ≥ 5 events/hour); 3) received treatment with CPAP or MAA; 4) received two PSG recordings, one without treatment and one with treatment; 5) the PSG recordings had bilateral masseter muscle electromyographic (EMG) traces [17]. Individuals were excluded: 1) with < 4 h of sleep during the recording; 2) with missing data on electroencephalography or masseter EMG; 3) with other sleep-related breathing disorders (e.g., asthma, chronic obstructive pulmonary disease), neurological disorders (e.g., epilepsy), or sleep-related movement disorders except for SB (e.g., periodic limb movement disorder); 4) received other OSA treatments, such as bariatric surgery, oral and maxillofacial surgery, and positional therapy.

### Polysomnography and scoring criteria

Full night PSG recordings were performed by a portable compact PSG system (SOMNOscreen Plus, SOMNOmedics GmbH, Germany). The PSG system consisted of electroencephalography (F4C4, C4O2, F3C3, C3O1), electrooculogram (E1M2, E2M1), electrocardiogram, EMG (bilateral masseter muscles, anterior tibialis, mentalis and submentalis), pressure airflow, abdominal/thoracic respiratory effort, oxygen saturation, plethysmograph, heart rhythm, and sleep position. The mounting was performed by certified sleep technicians at the Department of Clinical Neurophysiology, OLVG West, Amsterdam, the Netherlands.

PSG recordings were analyzed offline using Domino software (SOMNOmedics GmbH, Germany). Sleep stages and respiratory events (viz., apnea, hypopnea, and respiratory effort-related arousal) were scored manually by sleep technicians from OLVG according to the American Academy of Sleep Medicine (AASM) scoring manual of sleep and associated events [18]. Sleep arousals were scored by two of us (D.L. and B.K.), following the scoring rules of AASM [18]. Sleep arousals were classified as respiratory arousals (RAr) or non-respiratory arousals (nRAr) [19]. Arousals occurring at the termination of respiratory events were defined as RAr, while those without preceding respiratory events were defined as nRAr.

The masseter EMG signals were filtered according to the AASM scoring manual (50 Hz notch; 10 Hz high pass; 100 Hz low pass) [18]. RMMA was scored according to previously reported criteria [17]. Each RMMA burst must exceed twice the amplitude of background EMG and be present simultaneously on the bilateral masseter muscles EMG traces. RMMA bursts occurring at an interval shorter than 3 s were regarded as a single episode. RMMA episodes were classified into three subtypes: phasic RMMA (three or more continuous RMMA bursts that are 0.25-2 s in the duration), tonic RMMA (one or more RMMA bursts ≥ 2 s), and mixed RMMA (if both phasic and tonic RMMA bursts are present within an episode). In addition, RMMA was considered to be related to arousal (RMMA-RAr or RMMA-nRAr) when they occurred within 5 s of an arousal [20].

### Statistical analysis

The number of RMMA episodes was transformed into an index, defined as the number of events per hour of sleep. Individuals with an RMMA index of at least two episodes per hour of sleep were diagnosed with SB [17]. Individuals with concomitant OSA and SB were included in a subgroup for subgroup analysis. As the treatment durations of OSA therapies were not recorded in all participants’ medical history, this parameter was represented by the interval of the two PSG recordings for each patient.

The normality of outcome variables was assessed by using the Shapiro–Wilk test. Normally distributed data are presented as mean (standard deviation, SD). Non-normally distributed data are presented as quartiles (25%|median|75%). The comparisons of baseline characteristics of the two therapy groups, including sleep variables, respiratory variables, and treatment durations, were analyzed using the independent t-test (for normally distributed variables), Mann–Whitney U test (for non-normally distributed variables), or Chi-square test (for nominal variables).

Two-way repeated measures analyses of variance (ANOVA) were applied to analyze the mean difference of RMMA variables (i.e., RMMA index, RMMA-RAr index, and RMMA-nRAr index) separately for within-subjects factor (with versus without therapy) and to assess the interaction effect between the two factors (treatment effect of CPAP versus that of MAA). The baseline characteristics that showed significant differences between the two therapy groups were included as covariates of the two-way repeated measures ANOVA. Further, when CPAP and MAA showed significantly different effects on the RMMA variables, paired t-test or Wilcoxon signed-rank test were performed for each therapy group separately. The statistical analyses were performed in the total group as well as in individuals with comorbid SB. The significance level α of all statistical tests was set at 5%. All statistical analyses were performed using the SPSS statistics software package (version 26.0, SPSS Inc., Chicago, IL).

## Results

**Fig.1:**
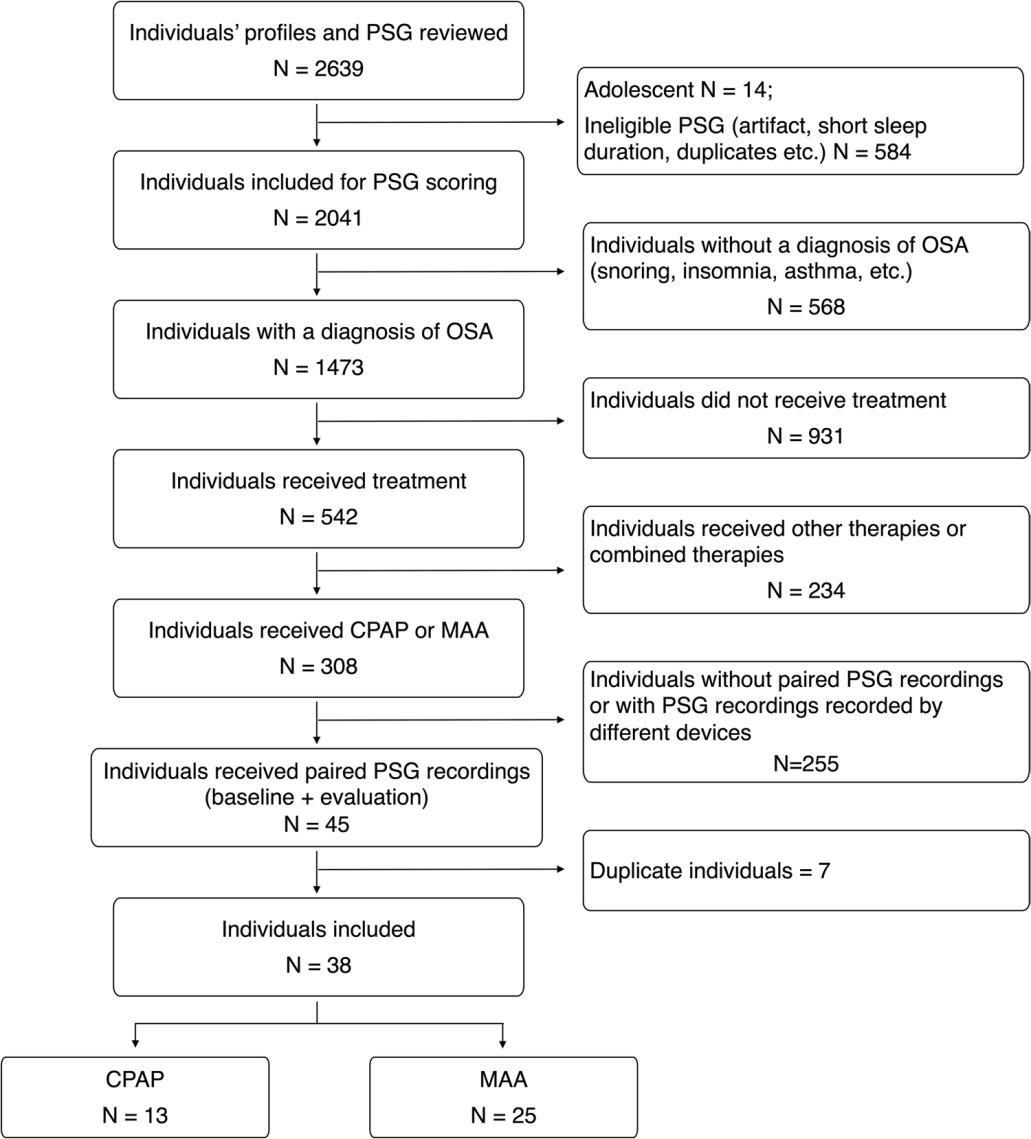
Flow diagram of participants in this study. PSG: polysomnography; OSA: obstructive sleep apnea; CPAP: continuous positive airway pressure; MAA: mandibular advancement appliance

Figure 1 shows a flow diagram of participants. We reviewed the medical history and PSG recordings of 2639 patients. According to the inclusion and exclusion criteria, 38 patients with OSA (32 men) were included in this study. Among them, 6 were diagnosed with mild OSA, 23 with moderate OSA, and 9 with severe OSA. Their median AHI was 23.7 events/hour (range from 8.6 to 75.9); their mean age was 52.6 ± 10.6 years; and their mean BMI was 25. 4 ± 6.3 kg/m^2^. Patients’ treatment duration varied from 75 to 598 days, with a median of 229 days. Thirteen individuals received CPAP and 25 received MAA therapy. In addition, 21 individuals (17 men) were diagnosed with comorbid SB (6 received CPAP and 15 received MAA).

Sleep variables, including total sleep time, sleep efficiency, and percentages of sleep positions, did not change significantly with CPAP or MAA therapy (see Table 1). Both therapies reduced the AHI and ODI significantly (both *P* < 0.01). With regard to arousal-related variables, CPAP significantly decreased the total arousal index and RAr index (both *P* < 0.01), while MAA only reduced the RAr index (*P* = 0.006). Both therapies did not affect the nRAr index (*P* > 0.05).

**Table 1:** Characteristics and polysomnographic variables of two therapy groups

	CPAP ^a^ (*N* = 13)		P ^b^	MAA ^a^ (*N* = 25)		P ^b^	P ^c^
	Without	With		Without	With		
Age, years	50.8 (11.3)	–	–	53.6 (10.3)	–	–	0.447
Gender, female/male	0/13	–	–	6/19	–	–	0.054
BMI	27.4 (4.5)	–	–	27.5 (3.9)	–	–	0.942
Total sleep time, h	6.9 (0.9)	6.4 (0.7)	0.113	6.4|6.7|7.3	6.3|6.8|7.9	0.627	0.878
Sleep efficiency, %	88.6 (8.2)	86.3 (9.9)	0.571	84.8|90.8|95.1	86.6|90.8|94.6	0.946	0.939
N1, %	5.7 (3.7)	4.4 (2.8)	0.291	2.3|4.1|6.4	2.5|4.3|7.3	0.798	0.433
N2, %	43.7 (12.1)	41.7 (7.4)	0.500	45.2 (11.3)	46.4 (9.8)	0.617	0.706
N3, %	19.2 (6.0)	22.0 (7.7)	0.256	18.2 (8.3)	16.9 (6.7)	0.516	0.695
REM, %	18.4|19.6|21.6	14.2|14.9|24.0	0.345	17.3 (7.3)	19.2 (6.6)	0.249	0.214
Supine, min	32.3|104.4|125.1	59.5|87.5|174.0	0.421	68.4|132.4|173.0	12.5|101.1|258.7	0.443	0.236
Non-supine, min	297.2 (128.6)	264.2 (107.7)	0.244	252.4 (104.6)	277.8 (139.3)	0.311	0.640
Total arousal, N/h	30.7 (12.1)	14.9 (8.0)	0.000*	25.5 (12.4)	21.7 (10.5)	0.152	0.222
RAr, N/h	8.9|12.7|17.1	0.0|0.2|0.9	0.001*	6.6|8.8|14.9	2.5|4.6|9.4	0.006*	0.249
nRAr, N/h	14.3 (6.5)	13.7 (7.8)	0.715	9.1|10.8|18.5	9.2|14.3|20.8	0.968	0.770
AHI, N/hour	23.5|24.4|31.3	0.3|0.7|4.0	0.001*	16.9|21.7|27.1	8.5|11.1|21.5	0.002*	0.103
ODI, N/hour	37.6 (19.1)	10.3 (7.5)	0.000*	18.7|25.6|32.7	12.4|14.7|28.2	0.003*	0.054
Treatment duration, days	146|209|232		–	155|252|318		–	0.286

^a^ Data are presented as mean (standard deviation) for normally distributed variables and as 25%|median|75% percentiles for non-normally distributed variables

^b^ Comparisons between PSG recordings without and with therapy were performed by paired t-test for normally distributed data, by Wilcoxon signed-rank test for non-normally distributed data

^c^ Comparisons of baseline characteristics between CPAP and MAA were performed by Independent t-test for normally distributed data, Mann–Whitney U test for non-normally distributed data, and by χ^2^ test for nominal variables

^*^ Statistically significant values at the 0.05 probability level

CPAP: continuous positive airway pressure; MAA: mandibular advancement appliance; BMI: body mass index; N1-3: non-rapid eye movement sleep stage 1–3; REM: rapid eye movement sleep stage; RAr: respiratory arousal index; nRAr: non-respiratory arousal index; AHI: apnea–hypopnea index; ODI: oxygen desaturation index

The baseline characteristics and PSG variables of participants are presented in Table 1. None of the variables differed significantly between the CPAP and MAA therapy groups. Therefore, no variable was taken as a covariate in ANOVA. Table 2 shows the values of RMMA variables in the total group and in the subgroup (i.e., OSA individuals with comorbid SB). Both in the total group and in the subgroup, the RMMA index decreased significantly with OSA therapies (*P* < 0.05 and 0.01, respectively). In addition, based on the interaction effect, the changes in the RMMA index between PSG recordings without and with therapy did not differ significantly between CPAP and MAA (*P* > 0.05).

**Table 2:** Effects of OSA therapies on sleep bruxism ^a, b^

	CPAP		MAA		Without vs. with therapy			Interaction effect (CPAP vs. MAA)	
Total group: OSA individuals (*N* = 38)									
	Without	With	Without	With	F (1, 36)	P	F (1, 36)		P
Individuals with OSA (*N* = 38)									
RMMA index	2.9 (2.8)	2.1 (2.0)	3.7 (3.2)	2.1 (2.8)	6.423	0.016*	0.646		0.427
RMMA-RAr index	1.6 (1.6)	1.7 (1.8)	1.6 (1.5)	0.7 (1.4)	16.571	0.000*	1.005		0.323
RMMA-nRAr index	1.0 (1.0)	1.7 (1.8)	1.7 (1.6)	1.2 (1.4)	0.141	0.710	8.240		0.007*
Subgroup: OSA individuals with SB (*N* = 25)									
	Without	With	Without	With	F (1, 23)	P	F (1, 23)		P
RMMA index	5.1 (3.0)	3.0 (2.7)	5.6 (2.8)	2.7 (3.4)	11.041	0.004*	0.323		0.577
RMMA-RAr index	2.8 (1.7)	0.1 (0.1)	2.5 (1.5)	1.0 (1.8)	17.262	0.001*	1.651		0.214
RMMA-nRAr index	1.5 (1.2)	2.5 (2.4)	2.6 (1.5)	1.5 (1.7)	0.023	0.882	9.233		0.007*

^a^ Two-way repeated measures analyses of variance was applied to analyze the difference between CPAP and MAA

^b^ Data are presented as mean (standard deviation)

^*^ Statistically significant values at the 0.05 probability level

OSA: obstructive sleep apnea; CPAP: continuous positive airway pressure; MAA: mandibular advancement appliance; RMMA: rhythmic masticatory muscle activity; RMMA-RAr: RMMA related to respiratory arousal; RMMA-nRAr: RMMA related to non-respiratory arousal

**Fig. 2:**
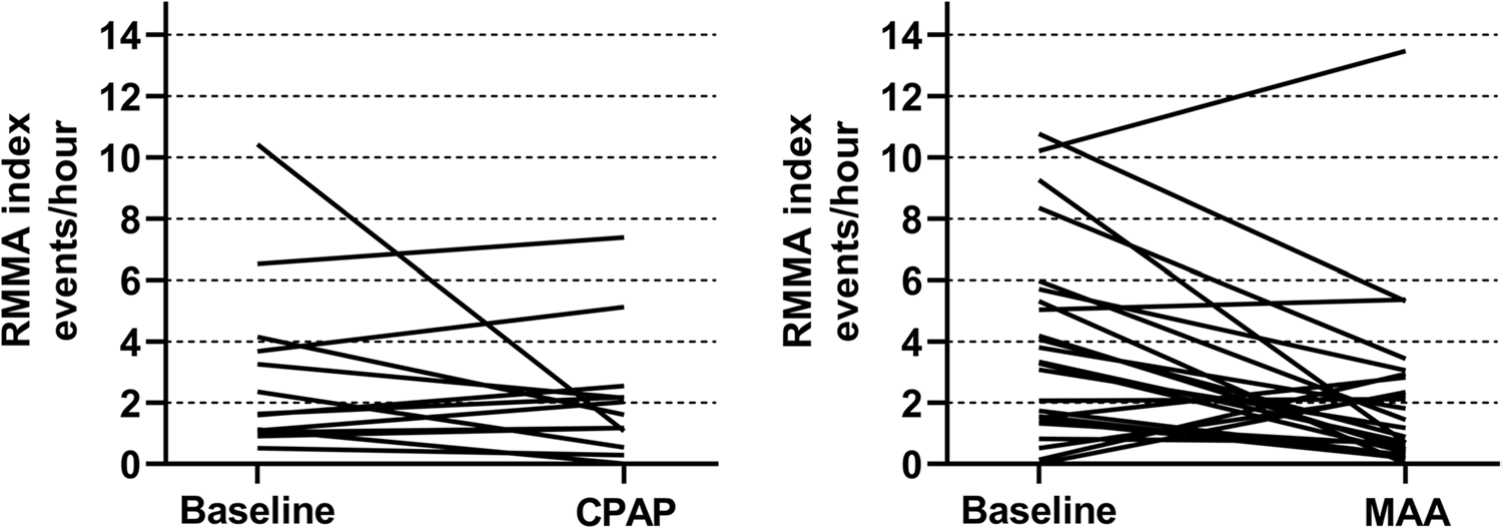
Individual values of RMMA indices in polysomnographic recordings without and with CPAP and MAA. RMMA: rhythmic masticatory muscle activity; CPAP: continuous positive airway pressure (*N* = 13); MAA: mandibular advancement appliance (*N* = 25)

Figure 2 shows the individual values of RMMA indices in PSG recordings with and without therapy. Among individuals who received CPAP (*n* = 13), six (46%) showed a decrease in the RMMA index, while seven (54%) showed an increase. For MAA, the RMMA index decreased in 17 individuals (68%), while it increased in seven individuals (28%), and one case (4%) showed no difference in the RMMA index. In OSA individuals with comorbid SB, the RMMA index decreased in four of the six cases who received CPAP (67%) and in 12 of the 15 cases who received MAA therapy (80%).

In addition, in the total group, the decrease of the percentage of the RMMA index was (-25%|52%|82%). For CPAP and MAA, it was (-34%|-13%|61%) and (-2%|59%|86%), respectively. The changes of the percentage of the RMMA index showed no significant difference between CPAP and MAA (*P* = 0.159). In individuals with OSA and comorbid SB, the decrease of the percentage of the RMMA index was (16%|61%|84%). For CPAP and MAA, it was (-13%|48%|77%) and (47%|62%|88%), respectively. The changes of the percentage of the RMMA index showed no significant difference between CPAP and MAA (*P* = 0.381).

During CPAP and MAA therapy, the RMMA-RAr index reduced significantly, and no significant difference in the changes of the RMMA-RAr index was found between the two therapies. The RMMA-nRAr index showed no significant changes in the total group and in the subgroup. However, CPAP and MAA showed a significantly different effect on the changes in the RMMA-nRAr index (*P* < 0.05). For this, a post-hoc analysis was conducted to analyze the effect of CPAP and MAA separately. In the total group, the RMMA-nRAr index increased significantly with CPAP treatment (Z = -2.027, *P* = 0.043), while it did not change significantly with MAA in situ (Z = 1.786, *P* = 0.074). To the contrary, in individuals with OSA and SB, CPAP did not affect the RMMA-nRAr index (T = -1.644, *P* = 0.161), but MAA decreased the RMMA-nRAr index significantly (Z = -2.385, *P* = 0.017).

## Discussion

Previous case reports showed that CPAP could relieve SB in cases with severe OSA, and several studies showed that MAA could do so in otherwise healthy individuals with SB [11–13, 21]. However, the effects of OSA therapies on SB has rarely been investigated in adults with OSA. Since SB could be primary, i.e., without clear cause, or secondary to other disorders [22], the underlying mechanism for the genesis of different phenotypes of SB might be different. Subsequently, the treatment effects of CPAP and MAA on SB may also vary depending on the characteristics of the population under investigation. Considering SB is highly prevalent in individuals with OSA (30% –50%) [10], it is clinically relevant to understand the effects of these therapies on SB in the OSA population.

As expected, both CPAP and MAA decreased the RMMA index in individuals with OSA as well as in those with comorbid SB. Therefore, we accepted our hypothesis for the primary aim that both CPAP and MAA will reduce the frequency of RMMA in individuals with OSA. These results are consistent with previous studies in individuals with primary SB [11, 13, 21]. Further, the results support that in individuals with comorbid OSA and SB, OSA therapies can improve both conditions [10]. Based on this, for individuals with concomitant OSA and SB, OSA therapies should be adopted in the first step. Then, sleep physicians should evaluate if the negative consequences of SB, such as jaw muscle pain, temporomandibular joint disorder or teeth wear, are still severe enough to necessitate a collaborative management by sleep physicians and dentists.

In addition, our results showed that there was no significant difference in the changes of the RMMA index between CPAP and MAA. This could be explained by several reasons. First, most of the participants in our sample were diagnosed with mild to moderate OSA. As reported in previous studies, CPAP and MAA did not show significant difference in the treatment of mild to moderate OSA [23, 24]. Thus, the possible difference between CPAP and MAA in the changes of RMMA that benefited from the improvement of OSA may not be present in adults with mild to moderate OSA. Further studies are recommended to confirm whether or not CPAP is more effective than MAA in relieving SB in individuals with severe OSA. Second, although SB may be a motor response to sleep arousals during sleep [25], some studies demonstrated that sleep arousal only acts as a permissive window for the occurrence of SB rather than a trigger [26]. Therefore, the reduction of sleep arousal may not have a linear relationship with that of RMMA. Based on this, although CPAP reduced more RAr than MAA (Table 1), it is possible that CPAP did not show a better effect on reducing the RMMA index than MAA. In addition, MAA has been reported to be able to relieve SB in individuals with SB but without OSA, implying that MAA may have other mechanisms for reducing RMMA compared with CPAP, e.g., by novelty effect of MAA, reducing the contractile properties of masseter muscles when the mandible is advanced by MAA, reducing mandibular movement freedom, and inducing masticatory muscle pain which then decreases RMMA [27].

Although both CPAP and MAA showed a consistent effect on the total RMMA index and RMMA-RAr index, the two therapies showed different effects on the RMMA-nRAr index. In the total group, the RMMA-nRAr index increased with CPAP therapy (*P* = 0.043), but no change was found with MAA in situ (*P* = 0.074). In individuals with OSA and SB, CPAP did not affect the RMMA-nRAr index (*P* = 0.161), while MAA decreased the RMMA-nRAr index significantly (*P* = 0.017). In short, MAA seems to have an additional effect on RMMA related to non-respiratory arousal compared with CPAP. This is consistent with the abovementioned notion that there are multiple mechanisms of MAA for reducing RMMA.

It is noteworthy that the effect of OSA therapy on SB seems to vary at an individual level. In the present study, around 60% of individuals with OSA (6/13 in CPAP, 17/25 in MAA) showed a decrease in the RMMA index with OSA treatment. In addition, the percentage of the changes of the RMMA index with OSA therapies among individuals had a wide range. This phenomenon has been reported in previous study [28]. There may be several reasons for such variances. First, previous study demonstrated that RMMA index has a time-variant nature across nights (22%-37%) [29]. Thus, the changes of the RMMA index in this study may be partially due to the natural fluctuation of RMMA. Second, although anterior tongue positions can reduce masticatory muscle activity and improve upper airway patency under CPAP and MAA, they may also increase masticatory muscle activity in some cases [30]. Third, the treatment durations of patients in this study presented a large variance, which may contribute to the individual differences, especially when considering the possible novelty effects of OSA therapies. Last but not least, according to the etiology of SB, SB could be primary or secondary related to other medical conditions [22]. Thus, we can speculate that RMMA episodes may have mixed pathogenesis both between and within individuals. Based on this, combined therapies, involving primary therapy for OSA and supplementary therapies for the remaining signs or symptoms of SB, would be better to improve patients’ quality of life.

### Limitations

Several limitations should be taken into consideration when interpreting the results of this study. First, since this is not a randomized controlled trial (RCT), the comparison of the effects of CPAP and MAA on SB may be biased. Nevertheless, our results show that both CPAP and MAA can effectively relief SB in most cases. Future studies are recommended to adopt RCT design to compare the effects of OSA therapies, thus supplying solid evidence for treatment of cases with concomitant OSA and SB. Second, this study did not involve a healthy control group so that the observed effect of CPAP and MAA on SB cannot be definitively attributed to the therapies themselves. Third, a limited sample was included in this study, especially for the CPAP group. Besides, as shown in Fig. 2, two cases showed substantial decreases with CPAP and MAA therapies, which could be considered as outliers. However, possibly, these purported outliers may be common observations in a large sample study. This could be supported by the large interindividual discrepancy in the effects of CPAP and MAA on SB that was presented in this study and by previous case reports which showed that SB episodes could disappear completely with CPAP treatment [11, 12]. For that reason, we did not remove these cases from analysis. Fourth, the signs and symptoms of SB before and after treatment, the actual treatment durations, patients’ adherence, and the decision-making process of OSA therapy for each patient, were not collected at the moment of data collection. Future RCTs with large samples are needed to confirm our findings in individuals with OSA and to compare the effects of different treatment modalities with different configurations.

## Conclusions

Within the limitations of this study, we concluded that both continuous positive airway pressure and mandibular advancement appliance significantly reduce sleep bruxism in individuals with obstructive sleep apnea and in those with comorbid sleep bruxism. No significant difference regarding the effects on sleep bruxism was found between the two therapies. In addition, the two therapies can reduce sleep bruxism episodes related to respiratory arousal but may have different effects on those related to non-respiratory arousal. It is noteworthy that the interindividual differences in the effects of both therapies on sleep bruxism are large.

## Data Availability

The data underlying this article cannot be shared publicly due to the privacy of individuals that participated in the study. The aggregate data will be shared on reasonable request to the corresponding author.
